# HPV8 Reverses the Transcriptional Output in Lrig1 Positive Cells to Drive Skin Tumorigenesis

**DOI:** 10.3390/cancers14071662

**Published:** 2022-03-25

**Authors:** Adnan Shahzad Syed, Gian Paolo Marcuzzi, Daliborka Miller-Lazic, Jochen Hess, Martin Hufbauer, Baki Akgül

**Affiliations:** 1 Faculty of Medicine and University Hospital of Cologne, Institute of Virology, University of Cologne, Fürst-Pückler-Str. 56, 50935 Cologne, Germany; adnan.syed@uk-koeln.de (A.S.S.); gian.marcuzzi@uk-koeln.de (G.P.M.); miller-lazic@gmx.de (D.M.-L.); martin.hufbauer@uk-koeln.de (M.H.); 2 Research Group Molecular Mechanisms of Head and Neck Tumors, German Cancer Research Center (DKFZ), 69120 Heidelberg, Germany; jochen.hess@med.uni-heidelberg.de; 3 Department of Otolaryngology, Head and Neck Surgery, Heidelberg University Hospital, 69120 Heidelberg, Germany

**Keywords:** human papillomavirus type 8 (HPV8), transgenic mouse models, skin tumour development, differentially regulated genes

## Abstract

**Simple Summary:**

Human papillomavirus (HPV) of genus beta (betaHPV) infects cutaneous epithelia and contributes to skin carcinogenesis, particularly in immunosuppressed patients. The HPV8 transgenic mouse model serves as a model for betaHPV-induced skin tumorigenesis. These animals express the complete early genome region of HPV8 under the control of the keratin (K)-14 promoter (K14-HPV8-CER). Skin tumorigenesis in these mice is driven by Lrig1-positive stem cells. To understand the role of HPV8 gene expression in Lrig1+ keratinocytes, we determined the transcriptional network in K14-HPV8-CER skin tumours and compared it to the already known pattern in Lrig1 stem cells. We showed that HPV8 differentially regulates 397 cellular genes in skin tumours and subverts the expression pattern of 23 genes in Lrig1+ cells. This study identified gene targets of HPV8 and its upstream regulators, which may play an important role in HPV8 mediated skin tumorigenesis.

**Abstract:**

K14-HPV8-CER transgenic mice express the complete early genome region of human papillomavirus type 8 (HPV8) and develop skin tumours attributed to the expansion of the Lrig1+ stem cell population. The correlation between HPV8-induced changes in transcriptional output in the stem cell compartment remains poorly understood. To further understand the oncogenic pathways underlying skin tumour formation we examined the gene expression network in skin tumours of K14-HPV8-CER mice and compared the differentially expressed genes (DEG) with those of the Lrig1-EGFP-ires-CreERT2 mice. Here, we report 397 DEGs in skin tumours of K14-HPV8-CER mice, of which 181 genes were up- and 216 were down-regulated. Gene ontology and KEGG pathway enrichment analyses suggest that the 397 DEGs are acting in signalling pathways known to be involved in skin homeostasis. Interestingly, we found that HPV8 early gene expression subverts the expression pattern of 23 cellular genes known to be expressed in Lrig1+ keratinocytes. Furthermore, we identified putative upstream regulating transcription factors as well as miRNAs in the control of these genes. These data provide strong evidence that HPV8 mediated transcriptional changes may contribute to skin tumorigenesis, offering new insights into the mechanism of HPV8 driven oncogenesis.

## 1. Introduction

Cutaneous squamous cell carcinomas (cSCC) are the most frequent metastatic skin cancers, and their global incidence is constantly increasing. Chronic UV exposure is known to be the leading cause for the development of cSCC, a tumour entity which arises from precancerous lesions termed actinic keratoses. Recent epidemiological data strongly implicate, that human papillomaviruses (HPV) of genus betapapillomavirus (betaHPV) bear a co-factorial role in this process [1,2,3]. Infections of the skin with betaHPV generally occur early in childhood, when the virus becomes part of the microbiological skin flora. In immunocompetent individuals, persistent HPV infections of the skin are well controlled and mostly asymptomatic. However, betaHPV infections may result in pathogenic skin conditions in immunocompromised patients (e.g., immunosuppressed organ-transplant-recipients) and patients with the rare genetic disorder Epidermodysplasia Verruciformis (EV). Of note, immunosuppression results in higher betaHPV loads in the skin than what is observed in the healthy population [4,5,6]. This observation is consistent with the hypothesis that higher viral DNA loads may lead to enhanced activity of betaHPV, which in turn heightens the risk for the emergence of cSCC [1,7,8]. Such immunosuppressed individuals frequently exhibit rapidly developing field cancerization, predominantly at UV exposed body sites, leading to profound morbidity and high mortality rates [9,10].

Transgenic hemizygous mice expressing the complete early genome region (CER) of the betaHPV type 8 (HPV8) under the control of the human keratin-14 (K14)-promoter (referred to as K14-HPV8-CER) develop skin tumours, partially with moderate to severe dysplasia. In 6% of the animals, such cSCC develop spontaneously without any previous treatment with physical or chemical carcinogens [11,12,13,14]. In these mice UVA/B-irradiation and mechanical wounding of the skin reliably leads to tumour induction within 3 weeks. Tumour formation is accompanied by an upregulation of oncogenic miRNAs, such as miR-17-5p, -21 and -106a and a downregulation of the tumour-suppressive miR-155 and -206 [15]. In order to unveil changes in gene regulatory networks associated with the presence of the HPV8 oncogenes in the skin of transgenic mice, we previously published data from global gene expression profiling of the non-lesional K14-HPV8-CER mouse skin, which predicted altered transcript levels for only few cellular genes [14].

The bulge region of the hair follicle represents the natural reservoir of cutaneous papillomaviruses [16,17,18,19], and our group previously addressed the role of different hair follicle stem cells in HPV8-induced skin tumour development. We were able to show that skin thickening in K14-HPV8-CER mice is attributed to an expansion of the ‘Leucine Rich Repeats and Immunoglobulin Like Domains 1’ positive (Lrig1+) hair follicle stem cell population into the overlying infundibulum and adjoining interfollicular epidermis. These HPV8+/Lrig1+ cells showed an increased colony formation efficiency, which was consistent with an expansion of the stem cell population [10,20]. 

During steady-state homeostasis, Lrig1+ stem cells represent a distinct population of quiescent multipotent cells in the junctional zone in mouse hair follicles, contributing to all epidermal lineages. Following injury, these cells acquire lineage plasticity and form the interfollicular epidermis, sebaceous gland and hair follicle, entering the wound bed as a cohesive basal cell population [21,22,23]. 

Lrig1 has evolved as a tumour suppressor, which is feedback-induced by multiple oncogenic signals shown both in vitro and in vivo [24,25,26]. In vivo, Lrig1 controls proliferation by modulating the amplitude of ErbB signalling in proliferative Lrig1+ stem cells [27]. The activated proliferation signals in K14-HPV8-CER mice must therefore have either overcome Lrig1 inhibitory effects on ErbB signalling or utilized other signalling pathways. In a previous study, Page et al. (2013) assessed whether Lrig1-expressing cells were molecularly distinct from hair follicle stem cells and basal cells in the epidermis [21]. For their experiments the group created a mouse model with EGFP-IRES-CreERT2 inserted at the translational start site in exon 1 of the Lrig1 locus and used Lrig1-EGFP+ cells for transcriptional analysis. Here, 378 differentially expressed genes were found in Lrig1+ cells. Curiously, the Lrig1-positive stem cells from the upper junctional zone displayed a highly proliferative phenotype and contributed to either the infundibulum or sebaceous gland replenishment, independently from interfollicular epidermis. 

In the present study, we analysed the gene network in K14-HPV8-CER skin tumours to provide a more comprehensive understanding of the tumorigenic pathways underlying betaHPV mediated skin tumorigenesis, that may facilitate the identification of novel druggable targets. To this end, we analysed gene expression in skin tumours of K14-HPV8-CER mice, and compared the expression profile with that of published signature genes for Lrig1-EGFP+ cells in Lrig1-EGFP-ires-CreERT2 mice [21] to arrive at a deeper understanding regarding the effect of HPV8 early gene expression in the Lrig1+ compartment. 

## 2. Materials and Methods

### 2.1. Global Gene Expression Profiling and Data Analysis of K14-HPV8-CER Murine Skin

FVB/N wild-type (Charles River Laboratories, Sulzfeld, Germany) mice (*n* = 3) and the transgenic hemizygous FVB/N K14-HPV8-CER (FVB/N background) mouse line (*n* = 3) were used in this study. For global gene expression analysis healthy skin samples of FVB/N wild-type animals as well as spontaneously developed skin tumours of K14-HPV8-CER mice were collected. Isolated total RNA was stored at −20 °C in RNALater (Qiagen, Hilden, Germany). Global gene expression profiling of FVB/N wild-type skin as well as skin tumours of transgenic mice was performed using self-printed microarrays employing a set of 35,852 oligomers, which cover around 25,000 genes (MouseOligo Set Version 4.0, Operon, Cologne, Germany). Sample preparation, hybridization and data analyses were performed as previously described [28]. Prior to all experiments, total RNA was examined for integrity and purity using an Agilent RNA 6000 Series II Nano kit on a 2100 Bioanalyzer (Agilent, Santa Clara, CA, USA). Targeted mRNA was amplified in an in vitro transcription-based protocol and subsequently labelled with cyanine-3 and cyanine-5 in separate reactions. Each sample was then hybridized against universal reference on one array, having different fluorescent labels in a colour switch experiment. Hybridization processes followed by washing procedures were automatically conducted in a GeneTac chamber (Genomic Solutions, Ann Arbor, MI, USA) with hybridization time expanded to 23 h in order to increase signal intensity. Expression data comparing non-lesional FVB/N wild-type skin with K14-HPV8-CER skin tumours are presented in Table S1. 

### 2.2. UV Treatment of Murine Skin and RT-qPCR

FVB/N wild-type and K14-HPV8-CER mice were treated with UVA/B as previously described [13]. Skin biopsies were collected either from untreated mice or 24 days after treatment. RNA was isolated and reverse transcribed as described earlier [14]. The mRNA expression was quantified by RT-qPCR and normalized to the expression levels of HPRT1. Expression ratios were related to the mean of untreated FVB/N wild-type skin. The results are an average from *n* = 6 mice. Error bars represent the standard deviation. Statistical analyses were generated with Student’s *t*-test with * *p* < 0.05, ** *p* < 0.01 and *** *p* < 0.001. The following primers were used:

Klk6-fw: CTGAGGAGAATCCCAACTGC;

Klk6-rev: TGGTATCTGGGAAGTCACCAT;

Lcn2-fw: CTTCAAAATTACCCTGTATGGAAGA;

Lnc2-rev: GGGTGAAAGTTCCTTCAGT;

Fosl1-fw: GTGCAGAAACCGAAGAAAGG;

Fosl1-rev: TTCTCATCCTCCAATTTGTCG;

HPRT1-fw: CCTAAGATGAGCGCAAGTTGAA;

HPRT1-rev: CCACAGGACTAGAACACCTGCTAA.

### 2.3. IHC Staining

IHC staining of formalin-fixed and paraffin-embedded (FFPE) tissue sections of murine skin was carried out with anti-SOX9 (D8G8H, Cell Signaling, #82630, Frankfurt, Germany) and anti-YY1 antibodies (22156-1-AP, Proteintech, Rosemont, IL, USA), using the 3, 3′-diaminobenzidine (DAB) peroxidase substrate kit according to manufacturer’s instructions (Vector Laboratories, Burlingame, CA, USA). Staining specificity was confirmed with IgG isotype control antibodies (data not shown). Stained slides were scanned with the VENTANA DP 200 Slide Scanner (Roche, Mannheim, Germany) and were analysed with QuPath 0.3.2 software.

### 2.4. Dataset from Lrig1-EGFP-Ires-CreERT2 Mice

Lrig1-EGFP-IRES-CreERT2 mice were created via knock-in of an EGFP-ires-CreERT2 cassette into the endogenous Lrig1 locus in C57Bl/6 embryonic stem cells. For global gene-expression profiling, RNA from skin keratinocytes was isolated, pre-amplified (Ovation RNA Amplification System, NuGEN, Leeds, UK) and then hybridized to MouseWG-6 v.2 BeadChips (Illumina) [21].

### 2.5. Data and Statistical Analysis

#### 2.5.1. Differentially Expressed Genes

The raw data were analysed using normalization and log transformation. Differentially expressed genes were identified using a 2.5-fold change threshold and *p* < 0.001 was considered to indicate a statistically significant difference.

#### 2.5.2. Gene Ontology (GO) and Kyoto Encyclopedia of Genes and Genomes (KEGG) Pathway Enrichment Analyses

The online tool ShinyGO v0.741 (http://bioinformatics.sdstate.edu/go/) (accessed on 1 December 2021) was used for GO enrichment, utilizing category classes such as biological process, cellular component and molecular function, followed by KEGG analysis (https://www.genome.jp/kegg/) (accessed on 1 December 2021). For protein pathway and functional enrichment analyses, gene names were given as input and the *p*-value cut-off [False Discovery Rate (FDR)] was set to <0.05 to extract the 20 most significant pathways.

#### 2.5.3. Protein-Protein Interaction (PPI) Network Analysis

The protein–protein interaction network was constructed using the STRING online webserver (https://string-db.org/), (accessed on 1 December 2021) which predicts functional interactions of proteins by integrating data from published as well as predicted methods. Furthermore, to construct the PPI network all active interaction database sources were applied and an interaction score of >0.4 was used. Genes with an interaction score >5 were defined as hub genes in the regulatory network.

#### 2.5.4. Transcriptional Factor Enrichment Analysis

We performed transcriptional factor binding enrichment analyses using five TFBS databases oPOSSUM3.0 (http://opossum.cmmt.ubc.ca/oPOSSUM3/about.html) (accessed on 1 December 2021), ChEA3 (https://maayanlab.cloud/chea3/) (accessed on 1 December 2021), ENCODE, TRANSFAC/JASPER (https://jaspar.genereg.net/) (accessed on 1 December 2021) and TRUST (https://www.grnpedia.org/trrust/) (accessed on 1 December 2021). Furthermore, an overlap between transcription factors was performed and transcription factors present in three or more databases were selected. 

## 3. Results

### 3.1. Characterization of Global Gene Expression in Skin Tumours of K14-HPV8-CER Mice

**Figure 1:**
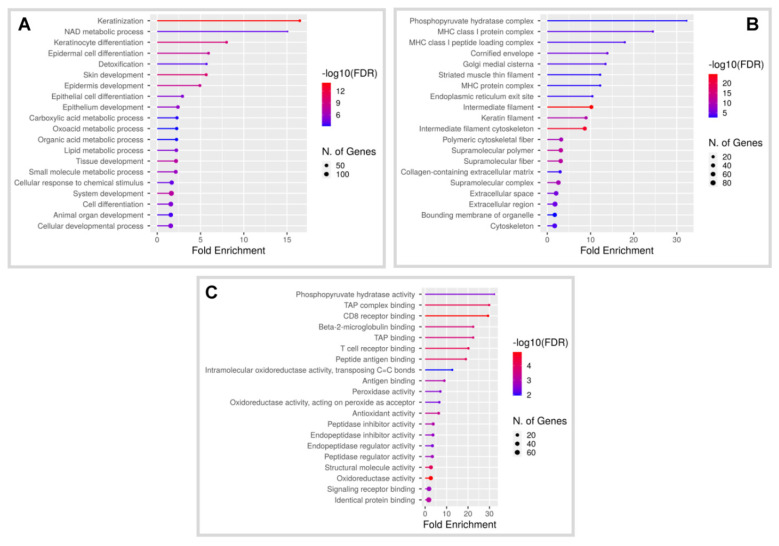
Gene ontology (GO) enrichment of DEGs in skin tumours of K14-HPV8-CER mice compared with non-lesional FVB/N wild-type skin. The GO categories (**A**) Biological process, (**B**) Cellular component and (**C**) Molecular function categories are shown (fold enrichment: high (red), low (blue)).

To further understand the underlying oncogenic mechanisms of HPV8-driven skin tumorigenesis, we used an Affymetrix microarray-based approach to determine global differences in cellular gene expression in K14-HPV8-CER skin tumours compared with that of non-transgenic mice of the same genetic background. Biopsies and total RNA were obtained from n = 3 mice in each group. Transcripts with a fold change of ≥2.5 and ≤−2.5 and *p*-value ≤ 0.001 (cut-off threshold) were treated as significantly altered. We detected a total of 397 differentially expressed genes (DEG) of which 181 were upregulated and 216 were downregulated (Table S1). To gain insight into the biological processes and pathways, we used the ShinyGO v0.741 software to interrogate Gene Ontology (GO) categories, thereby identifying biological processes altered by simultaneous expression of HPV8 early proteins. The top enriched GO terms for the DEGs, sorted by fold-change enrichment, are related to biological processes and cellular component such as ‘keratinization’, ‘NAD metabolic process’, ‘keratinocyte differentiation’, ‘epidermal cell differentiation’ and ‘keratin/intermediate filament’ (Figure 1A,B). The top GO molecular functions were found to be associated with sphingolipid metabolism, pyruvate hydratase activity and T-cell receptor binding (Figure 1C). Moreover, PPI interaction networks for up- and downregulated genes were created and are shown in Figures S1 and S2.

**Figure 2:**
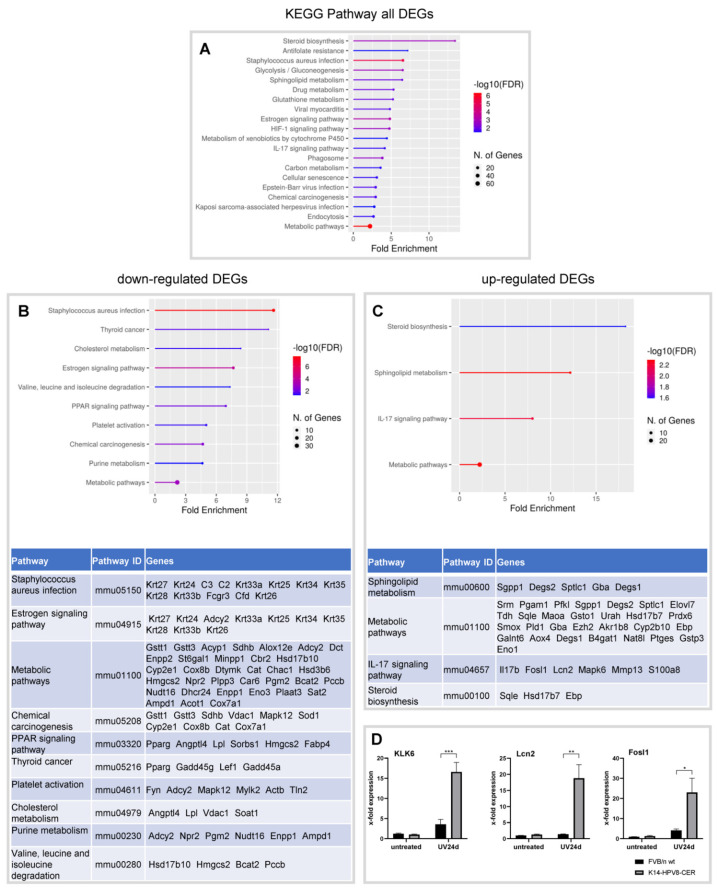
KEGG enrichment analysis for DEGs in skin tumours of K14-HPV8-CER mice. (**A**) KEGG enrichment analysis for all DEGs; KEGG enrichment analysis for only (**B**) downregulated and (**C**) upregulated DEGs. (**D**) KLK6, Lnc2 and Fosl1 mRNA expression is enhanced in UV-induced skin tumours in K14-HPV8-CER mice. Statistical analysis was generated with Student’s *t*-test with * *p* < 0.05, ** *p* < 0.01 and *** *p* < 0.001.

The KEGG pathway analysis pointed to four upregulated and 10 downregulated pathways. The analysis for downregulated DEGs pointed towards an enrichment of genes associated with a staphylococcus aureus infection. Upregulated pathways were mainly associated with sphingolipid metabolism and the IL-17 signalling pathway (Figure 2). Next, RT-qPCR was performed to validate the differential expression of some DEGs. As shown in Figure 2D, mRNA levels of KLK6, LCN2 and FOSL1 were found to be significantly overexpressed in UV-induced skin tumours in K14-HPV8-CER mice compared to FVB/N wild-type skin, collected 24d after irradiation. These data significantly strengthen the robustness of the microarray experiments. 

### 3.2. Identification of a Common Gene Set in Lrig1-EGFP-IRES-CreERT2 and K14-HPV8-CER Mice

**Figure 3:**
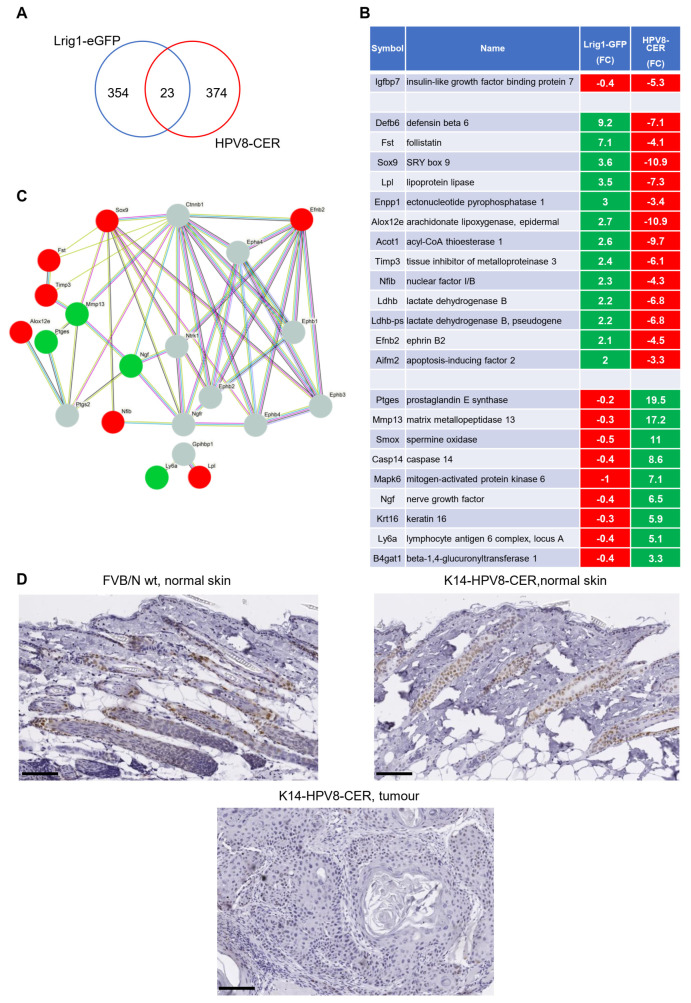
Comparison of DEGs differentially expressed in K14-HPV8-CER and Lrig1-EGFP-ires-CreERT2 mice. (**A**) Venn diagram showing the overlap of DEGs found in skin tumours of K14-HPV8-CER and the skin of Lrig1-EGFP-ires-CreERT2 mice. (**B**) Heat map of 23 genes identified by Venn analysis (green: upregulated genes, red: downregulated genes). (**C**) The protein–protein interaction (PPI) network plot for the 23 genes using STRING database (code colour: green, upregulated; red, downregulated; grey, database predicted interactors). Line colour indicates the type of interaction evidence e.g., known interaction, or predicted interaction. (**D**) Representative immunohistochemical staining for SOX9 in FVB/N wild-type, K14-HPV8-CER non-lesional skin as well as K14-HPV8-CER skin tumour. Staining revealed strong expression in FVB/N wild-type and non-lesional K14-HPV8-CER skin. SOX9 was found to be almost completely absent in K14-HPV8-CER tumours (scale bar: 100 μm).

Page et al. (2013) generated a mouse model with EGFP-IRES-CreERT2 inserted at the translational start site in exon 1 of the Lrig1 locus and used Lrig1-EGFP+ cells for transcriptional analyses [21]. Here, 378 genes were found to be differentially expressed in Lrig1+ cells compared to Lrig1- cells. We next analysed how these alterations may contribute to initiation of skin tumour development in K14-HPV8-CER mice. To this end, we compared our gene list with that of Page et al., which led to the identification of 23 overlapping genes (Figure 3A). Interestingly, we observed that, in the skin of K14-HPV8-CER mice, the expression patterns of 22 out of 23 genes were completely reversed compared to the expression levels in Lrig1-EGFP+ cells. Collectively, these data point towards a crucial impact of HPV8 early proteins on global gene expression homeostasis, which may also have an effect of cell proliferation in Lrig1+ stem cells (Figure 3B). Considering that it was not possible to perform GO and KEGG pathway analyses with a gene set of only 23 genes, we therefore constructed a protein–protein-interaction (PPI) network to further decipher the functional interplay of these 23 genes. Using two more levels of protein interaction nodes we found that four out of the identified 23 genes, namely EFNB2, NGF, MMP13 and SOX9, were hub nodes known to interact with five or more genes. Two of these hubs (MMP13, NGF) were found to be upregulated and two (EFNB2, SOX9) to be downregulated by HPV8 (Figure 3C). Immunohistochemical staining for SOX9 revealed strong signals in the hair follicles of FVB/N wild-type skin and non-lesional transgenic skin. However, SOX9 staining was discovered to be almost absent in K14-HPV8-CER tumours (Figure 3D), which is in line with the gene expression values.

### 3.3. Identification of Potential Upstream Transcriptional Regulators

**Figure 4:**
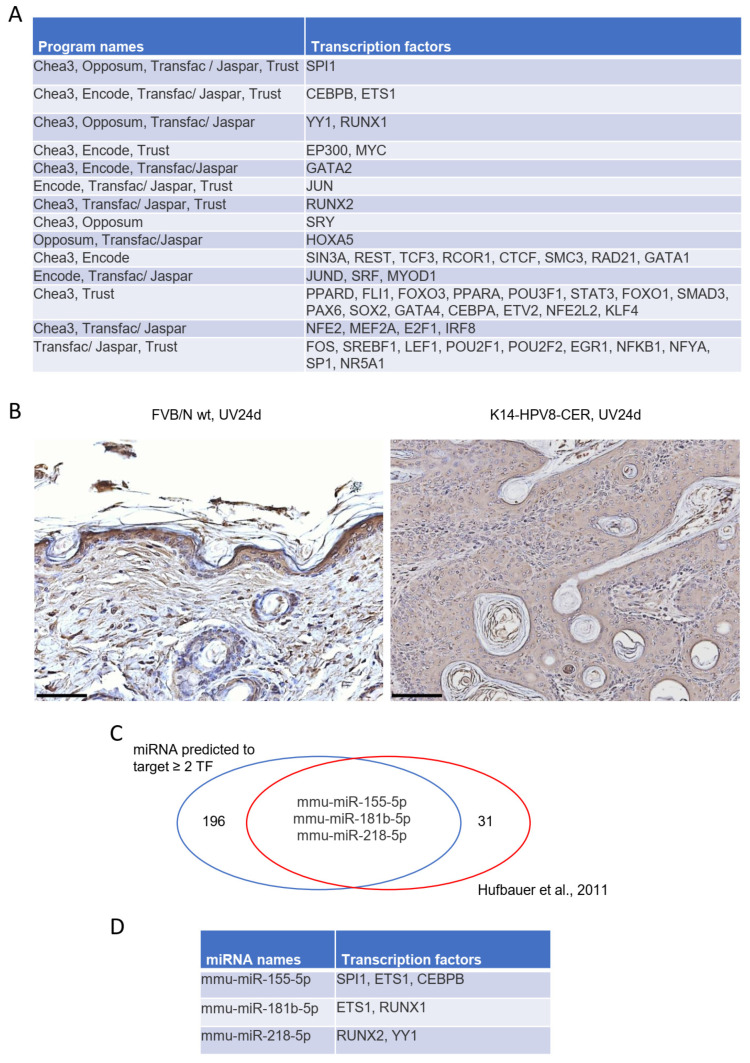
Identified potential transcription factors (TFs) for regulation of 23 DEGs. TFs prediction was performed using the online tools Chea3, Encode, Opposum, Transfac/Jasper and Trust. (**A**) List of TFs predicted by at least three different programs. (**B**) Representative YY1 staining of FVB/N wild-type skin (scale bar: 50 μm) and a skin tumour of a K14-HPV8-CER mouse (scale bar: 100 μm) taken 24 days post UV treatment. (**C**) Venn plot of miRNAs predicted to target ≥ 2 TFs and miRNAs previously shown to be deregulated in skin tumours of K14-HPV8-CER mice [15]. Analysis led to the identification of three miRNAs, namely mmu-miR-155-5p, mmu-miR-181b-5p and mmu-miR-218-5p to be upstream regulators. (**D**) The TFs as targets of these miRNAs are listed.

We next performed in silico analyses using the online tools CHEA3, OPPOSUM, TRANSFAC/JASPER and TRUST to predict transcription factors regulating these differentially expressed genes. These analyses led to the prediction of SPI1, CEBP/beta, ETS1, YY1, RUNX1, EP300, MYC, GATA2, JUN and RUNX2, which were all predicted by at least three of the above listed online tools to be involved in regulating the above-mentioned transcription factors (Figure 4A). As YY1 had been one of the predicted transcription factors, we analysed K14-HPV8-CER skin tumours for total levels of YY1 by immunohistochemistry. Strong nuclear YY1 expression could be detected in FVB/N wild-type skin. In basal keratinocytes of K14-HPV8-CER skin tumours, we observed no or only weak staining for YY1. In suprabasal cells, YY1 was found predominantly in the cytoplasmic compartment indicating disruption of YY1 localization in the presence of HPV8 early proteins (Figure 4B). 

In order to determine whether miRNAs can be linked to DEGs, which may play a central regulatory role controlling these tentative transcription factors, we first determined the miRNA–target pairs using the online tool MIRDB (Table S2), and then generated Venn diagrams to identify unique miRNAs already known to be differentially regulated in K14-HPV8-CER mice [15]. Interestingly, these analyses led to the identification of three miRNAs, namely mmu-miR-155-5p, mmu-miR-181b-5p and mmu-miR-218-5p (Figure 4B), which are all known to target SPI1/ETS1/CEBP/beta, ETS1/RUNX1 and RUNX2/YY1, respectively (Figure 4C,D).

## 4. Discussion

Tissue homeostasis is controlled by a complex interplay of both proliferative signalling and anti-proliferative mechanisms. An imbalance in this homeostasis can lead to tumorigenesis, occurring when oncogenic signalling overrides tumour-suppressive mechanisms. In order to extend our knowledge on these oncogenic effects mediated by HPV8, we used our K14-HPV8-CER mouse model to study the underlying molecular mechanisms leading up to betaHPV-driven skin carcinogenesis. The lack of in-depth knowledge regarding the pathophysiology of betaHPV mediated keratinocyte transformation remains a limiting factor in our understanding of the exact oncogenic mechanisms. Here, we were now able to identify gene clusters differentially expressed in K14-HPV8-CER skin tumours compared to FVB/N wild-type skin. Gene expression analyses performed in this study strongly indicate that HPV8 early gene expression in the murine skin changes gene expression involved in epidermal development and keratinization, a process which was expected due to the known activities of HPV early proteins on the epidermal differentiation program [29,30,31,32]. Furthermore, pathways associated with sphingolipid metabolism and regulation of immunological processes seem to also be hijacked by HPV8 to drive tumorigenesis. 

Moreover, our team showed that tumour formation in K14-HPV8-CER mice is associated with an expansion of the Lrig1+ stem cell population [10,20]. A comparison of our DEG list with that of Page et al. led to the identification of 23 genes known to be physiologically expressed in Lrig1+ stem cells. Therefore, these genes might be crucial for maintenance of skin homeostasis. 

More significantly, we found that transcription factor SOX9 was a hub gene that takes part in a diverse range of biological events and is downregulated by HPV8. It is known that SOX9 is positively regulated by β-catenin, whereas knockdown of β-catenin resulted in downregulation of SOX9 [33]. We could already show that the membrane-tethered inactive form of β-catenin becomes elevated in the presence of HPV8-E7, whereas we found no β-catenin activity within the canonical Wnt signalling pathway [34]. The importance of β-catenin in skin cancer is supported by the fact that epidermis-specific ablation of the β-catenin gene leads to abrogation of cancer initiating cells and tumour regression [35]. It is therefore tempting to speculate that HPV8 mediated downregulation of SOX9 may have resulted by targeting β-catenin localization.

We further show that SPI1, CEBP/beta, ETS1 (proposed by four different search programs), YY1, RUNX1, EP300, MYC, GATA2, JUN and RUNX2 (proposed by three different programs) are the proposed top ranked upstream transcription factors controlling the expression of the aforementioned 23 genes and may therefore represent key regulatory nodes of HPV8-induced skin tumorigenesis. CEBP/beta, p300 and YY1 are well known transcription factors targeted by HPV8 early proteins. The staining pattern for YY1 on the skin tumours of K14-HPV8-CER mice indicated that YY1 intracellular distribution is switched from nuclear to cytoplasmic in the presence of HPV8 early proteins and may play a role in HPV8-induced tumorigenesis. Interestingly, a similar dislocation of YY1 was previously reported in high-grade squamous intraepithelial lesions [36], which further underscores the importance of YY1 as a crucial transcriptional regulator targeted by HPV. HPV8-E2 and HPV8-E7 are known to bind to CEBP-beta [37,38], HPV8-E2 and HPV8-E6 to p300 [39,40], and thus may affect the transcriptional output of infected cells. SPI1 encodes PU.1, a transcription factor that has been found to be involved in the progression of various cancer types [41]. Both PU.1 and RUNX1 contain distal regulatory elements containing RUNX and ETS motifs, which indicates that both genes are autoregulated (reviewed in [42]). Furthermore, it needs to be mentioned that RUNX1 is known to directly promote proliferation of hair follicle stem cells and epithelial tumour formation in murine skin [43]. 

Our group recently showed that the inflammatory signal transducer and activator of transcription 3 (STAT3) pathway is highly active in K14-HPV8-CER murine skin and that keratinocyte-specific STAT3 heterozygosity interferes with skin tumour development [44]. Recently, it was shown that constitutively active STAT3 signalling significantly changes the behaviour of keratinocyte stem/progenitor cells residing in the hair follicle [45], and that disruption of STAT3 in keratinocytes compromises wound-healing and affects initiation and promotion of skin tumours [46,47]. STAT3 signalling is known to regulate miRNAs and to be regulated by miRNAs including miR-155-5p, miR-181b-5p and miR-218-5p [48,49]. Interestingly, SPI1, ETS1 and CEBP/beta are all known targets of miR-155 [50,51], with miR-155 mechanistically inhibiting PU.1 expression. All of this fits in with the previously described downregulation of miR-155 in skin tumours of K14-HPV8-CER mice [15]. It was recently demonstrated that miR-155 downregulates ErbB2 and suppresses ErbB2-induced malignant transformation of epithelial cells [52]. Considering that a key role of Lrig1 lies in the control of the Erb amplitude, it is tempting to speculate that the inhibition of miR-155 by HPV8 may abolish ErbB2 suppression and enhance the oncogenic functions of HPV8 early proteins. The other two identified miRNAs, namely miR-181b-5p and miR-218-5p, are both known to be involved in the control of keratinocyte proliferation, and, in the case of miR-218-5p also in hair follicle development [53,54]. The exact role of these miRNAs needs to be further researched in future studies. 

Another interesting observation is that, in Lrig1+ skin cells the IL17R / EGFR signalling axis links wound healing signalling to tumorigenic processes [55]. Since the KEGG pathway analysis of DEGs of K14-HPV8-CER skin tumours also suggest an enrichment of genes regulating IL-17 signalling comprising LCN2 and FOSL1, this strongly points to an important role of IL-17 signalling in HPV8-mediated tumorigenesis. Most interestingly, an immunosuppressive role for IL-17 signalling in HPV-associated epithelial hyperplasia was previously shown [56,57] and is known to promote tumour progression in human non-melanoma skin cancer cells [58]. Future studies are needed to determine whether blocking IL-17 signalling in persistent betaHPV infection may promote antiviral immunity and prevent tumorigenic processes. 

## 5. Conclusions

In conclusion, our analyses reveal a remarkable impact of HPV8 early proteins on cellular gene expression in the murine skin, hinting at a subversion of gene expression in Lrig1+ cells. Future studies will have to be conducted to specifically determine the impact of these changes on betaHPV-induced skin tumorigenesis to design and test new HPV-specific therapeutic strategies.

## Data Availability

The data presented in this study are available in this article (and Supplementary Materials).

## Supplementary Materials

The following supporting information can be downloaded at: https://www.mdpi.com/article/10.3390/cancers14071662/s1, Figure S1: The protein-protein interaction (PPI) network plot of 181 DEGs found to be upregulated in skin tumours of K14-HPV8-CER mice using STRING database. Figure S2: The protein-protein interaction (PPI) network plot of 216 DEGs found to be downregulated in skin tumours of K14-HPV8-CER mice using STRING database. Table S1: List of DEGs found in skin tumours of K14-HPV8-CER mice vs FBV/n wild-type skin. Table S2: List of predicted miRNAs targeting transcription factors using the web server mirdb.org.

## Abbreviations

cSCC: cutaneous squamous cell carcinoma; EGFP, enhanced green fluorescent protein; EV, Epidermodysplasia Verruciformis; HPV, human papillomavirus; HPV8-CER, complete early genome region of HPV8; ires, internal ribosome entry site; K14, keratin-14.
